# Global research status and frontiers on autophagy in hepatocellular carcinoma: a comprehensive bibliometric and visualized analysis

**DOI:** 10.1097/MS9.0000000000002711

**Published:** 2024-11-13

**Authors:** Yucheng Zhang, Tao Bao

**Affiliations:** a Changshu Hospital Affiliated to Nanjing University of Chinese Medicine, Suzhou, People’s Republic of China; b Chun’an County First People’s Hospital, Hangzhou, People’s Republic of China; c Medical College, Yangzhou University, Yangzhou, People’s Republic of China

*Dear Editor,*

As we all know that *Annals of Medicine and Surgery* is a prestigious and top academic journal in the field of surgery, we recently read with interest some of the high-quality studies in your journal, and one of the studies titled ‘Global research status and frontiers on autophagy in hepatocellular carcinoma: a comprehensive bibliometric and visualized analysis^1^’ caught our attention. This study used scientific literature search and econometric analysis to comprehensively search for studies in the field of autophagy in hepatocellular carcinoma, obtain annual publication data of these studies, summarize the countries, institutions, authors, and journals that have made significant contributions, and visualize keywords appearing in the studies, which provide a basis for observing current research hotspots and future research trends. The visualization of keywords in the research provides a basis for observing current research hotspots and future research trends, and provides an important reference for scholars who are new to the field or want to learn more about the field. As mentioned in the authors’ article, autophagy in hepatocellular carcinoma is a huge public health challenge that has received a great deal of attention in recent years, suggesting the importance of this study. However, after our careful reading, we have some suggestions about certain parts of this study that we would like to discuss with the authors and our readers, and we hope that the authors will share their views and ideas.

The first concern is that the author’s detailed search strategy is written in a supplemental table ‘TS=((primary hepatic cancer) OR (primary liver cancer) OR (hepatocellular carcinoma) OR (malignant hepatoma) OR (primary liver carcinoma)) OR (primary hepatic carcinoma) OR (HCC)) AND ((autophagy) OR (macroautophagy) OR (microautophagy) OR (chaperone-mediated autophagy) OR (mitophagy) OR (ferritinophagy) OR (aggrephagy) OR (lipophagy) OR (clockophagy) OR (nucleophagy) OR (xenophagy))’. With the above search strategy, we could not retrieve the citation record correctly, this is an invalid Boolean search strategy, after our careful observation, we found that this search strategy is missing some brackets and Boolean operators, so after we adjusted it, the possible search formula should be ‘TS=((primary hepatic cancer) OR (primary liver cancer) OR (hepatocellular carcinoma) OR (malignant hepatoma) OR (primary liver carcinoma) OR (primary hepatic carcinoma) OR (HCC)) AND TS=((autophagy) OR (macroautophagy) OR (microautophagy) OR (chaperone-mediated autophagy) OR (mitophagy) OR (ferritinophagy) OR (aggrephagy) OR (lipophagy) OR (clockophagy) OR (nucleophagy) OR (xenophagy))’. We believe that this issue may have been due to the authors mistakenly uploading a previously incorrect search formula when submitting the manuscript, and did not affect subsequent research.

The authors’ current study is about autophagy in hepatocellular carcinoma, and we know that the Web of Science Core Collection (WOSCC) is a collection of databases that integrates multiple domains, including the Science Citation Index Expanded (SCIE) for research in the natural sciences from 1999 to the present; SSCI (Social Science Citation Index) for research in the social sciences from 2005 to the present; and AHCI (Arts and Humanities Citation Index) for research in the arts and humanities from 2005 to the present. (SCIE) for research in the natural sciences from 1999 to the present; SSCI (Social Science Citation Index) for research in the social sciences from 2005 to the present; AHCI (Arts and Humanities Citation Index) for research in the arts and humanities from 2005 to the present; ESCI (Emerging Sources Citation Index) for emerging research resources in recent years; CCR-Expanded (Current Chemical Reactions) for the latest chemical reactions from 2019 to the present; and CCR-Expanded (Chemical Reactions) for the latest chemical reactions from 2019 to the present. Chemical Reactions); IC (Index Chemicus), which includes information on organic compounds from 1993 to the present. Due to the diversity of databases, applying multiple databases for literature search may lead to a loss of scientific validity and rigor in this study^2,3^, and as the authors’ topic of this study belongs to the field of natural sciences, it is inappropriate to incorporate so many databases in a single bibliometric study; therefore, we recommend that the authors use the SCIE database for the metrological analyses after giving due consideration to the authors. In addition, as far as we know, most of the bibliometric analyses will only choose an article for the analysis^4,5^, because the review paper is just a comprehensive discussion and analysis of the previous articles, so we suggest that the authors would be better off choosing the appropriate type of literature and a more precise description in order to improve the scientific and rigorous nature of the results of the study. The results of the search will still incorporate a high number of irrelevant literature, and authors should describe in detail how they screened and cleaned up the studies that were not relevant to this study, or summarize the articles that were included after eliminating the irrelevant literature and place them in the supplemental material, which can be easily accessible to subsequent readers.

Last but not least, the H-index is widely used to evaluate a researcher’s scholarly output and level of excellence and to quantify the researcher’s impact in the field as an independent individual; in short, the H-index means that at most H papers have been cited at least H times^6^. We highly recommend that the authors include this indicator in subsequent studies.

In conclusion, we congratulate all the authors for the successful completion of this bibliometric work, which is the first of its kind to reveal the hotspots and future research directions in the study of autophagy in hepatocellular carcinoma. In order to further improve the scientific validity and rigor of the study, we believe that these recommendations we present here can help the authors to build on their previous work in a more reliable and comprehensive manner, and that this work will help to provide a valuable perspective and inform the trajectory of subsequent studies.

## Ethical approval

This study did not involve patients.

## Consent

This study did not involve patients.

## Source of funding

This research was supported by the Postgraduate Research and Practice Innovation Program of Jiangsu Province (Grant. SJCX24_2351).

## Author contribution

T.B.: make a great contribution to the overall research, design, and conception; Y.Y.: write manuscripts for data integration processing; Y.Z.: data collection.

## Conflicts of interest disclosure

Other than the disclosed information, the authors have no other relevant affiliations or financial dealings with any organization or entity that has a financial interest or financial conflict with the topic or material discussed in the manuscript.

## Research registration unique identifying number (UIN)

Not applicable.

## Guarantor

Tao Bao was able to take full responsibility for the study and make all decisions.

## Data availability statement

Data used to support the results of this study are available from the corresponding author upon request.

## Provenance and peer review

This paper has not been published anywhere or is a repeat submission.
